# Case Report: *FGFR1* mutation and massive chromosome loss drive malignant transformation of low-grade gliomas

**DOI:** 10.3389/fonc.2025.1694881

**Published:** 2025-11-14

**Authors:** Ruze Tang, Yanming Chen, Dong Wan, Yongjun Xiang, Si Chen, Huafei Chen, Xiaoxiao Dai, Rong Rong, Sheng Xiao, Qing Lan, Hangzhou Wang, Shungen Huang

**Affiliations:** 1 Department of General Surgery, Children’s Hospital of Soochow University, Suzhou, China; 2 Department of Neurosurgery, The Second Affiliated Hospital of Soochow University, Suzhou, China; 3 Advanced Molecular Pathology Institute of Soochow University and SANO, Suzhou, China; 4 Suzhou SANO Precision Medicine Ltd, SANO Medical Laboratories, Suzhou, China; 5 Department of Neurosurgery, Children’s Hospital of Soochow University, Suzhou, China; 6 Department of Pathology, The Second Affiliated Hospital of Soochow University, Suzhou, China; 7 Department of Biological Sciences, Xi An Jiaotong-Liverpool University, Suzhou, China

**Keywords:** *FGFR1*, haploidy, glioma, malignant transformation, methylation

## Abstract

The mitogen-activated protein kinase (MAPK) signaling pathway plays roles in cell proliferation, differentiation, and apoptosis, all crucial for cellular transformation. It’s no surprise that MAPK alterations are prevalent in numerous tumors. Several critical genes in the MAPK signaling pathway, including *BRAF, FGFR*, and *NF1*, are mutated in brain tumors. For example, *FGFR1* mutation or rearrangement has been described in pilocytic astrocytoma, diffuse astrocytoma, and dysembryoplastic neuroepithelial tumor (DNT). These MAPK-activated brain tumors are benign and seldom progress to malignancies, with the mechanisms driving this rare transformation not yet fully understood. In this study, we present two cases of high-grade glioma characterized by a single activating mutation of *FGFR1* and massive chromosome loss (near-haploid genome). Similar haploidy is found in 3 additional high-grade astrocytoma by literature review, all harbor a single gene mutation in the MAPK pathway. We propose that the massive chromosome loss might serve as a significant mechanism contributing to the unusual malignant transformation of benign brain tumors activated by the MAPK pathway.

## Introduction

The fibroblast growth factor receptors (FGFR) family of receptor tyrosine kinases plays crucial roles in cell proliferation, apoptosis, and angiogenesis, and is frequently implicated in various types of tumors (1). Somatic *FGFR* alterations are predominantly found in low-grade astrocytomas and glioneuronal and neuronal tumors. *FGFR1* partial duplication involving the tyrosine kinase domain (*FGFR1* TKD) has been identified in pilocytic astrocytoma (WHO grade 1 tumor), diffuse astrocytoma (grade 2), dysembryoplastic neuroepithelial tumor (DNT) (grade 1), and diffuse low-grade glioma, MAPK pathway-altered (2). *FGFR1* hotspot mutations, including N546 and K656, are commonly observed in pilocytic astrocytoma and DNT (2). *FGFR2-CTNNA3* rearrangement is predominantly seen in polymorphous low-grade neuroepithelial tumor of the young (PLNTY) (grade 1) (3). *FGFR* alterations can also be observed in high-grade gliomas, with *FGFR3-TACC3* rearrangement frequently observed in glioblastoma (grade 4) (4). In low-grade tumors, *FGFR* alteration/activation often occurs as the sole genomic change, suggesting its role as an initiation event. However, in high-grade gliomas, *FGFR* activation is frequently associated with other genomic alterations. It remains inconclusive as to whether these high-grade gliomas have progressed from previous low-grade gliomas or if *FGFR* alterations are secondary changes.

In this study, we report two cases of high-grade glioma with *FGFR1* mutation, each characterized by additional massive chromosomal loss. Interestingly, near-haploid genomes were also observed in two anaplastic pleomorphic xanthoastrocytomas (A-PXA) (grade 3), both carrying germline *NF1* mutations (5). Furthermore, we identified a pilocytic astrocytoma with a sole *BRAF* duplication in the fourth ventricle of the brain, which developed drop metastasis in the spine and underwent a malignant transformation to high-grade astrocytoma with piloid features (HGAP, grade 3). A near-haploid genome was detected in the transformed HGAP (**Table 1** in press). Common features among these tumors include alterations in the MAPK signaling pathway genes (*FGFR1, NF1, BRAF*) and the presence of a near-haploid genome. The MAPK gene mutations are often associated with low-grade gliomas. We propose that massive chromosome loss, resulting in the simultaneous loss of multiple tumor suppressors, represents an important mechanism in tumor progression and malignant transformation for these typically benign brain tumors.

**Table 1:** Summary of 5 cases of high-grade glioma with both a haploid genome and a gene mutation in the MAPK signaling pathway.

*MAPK* path gene mutated	Additional genomic profiling	Histology diagnosis	Age (years)	PMID
*NF1*	28,Y,+4,+5,+7,+12,+16	Anaplastic pleomorphic xanthoastrocytomas	Adult (≥18)	32619305
*NF1*	29,X,+4,+5,+7,+12,+16,+17q	Anaplastic pleomorphic xanthoastrocytomas	Adult	32619305
*FGFR1*	26,X,+4,+7,+12 (duplicated genome)	High-grade glioma, NEC	63	This study(Case 1)
*FGFR1*	28,-X,+6,+10,+11,+12,+21,+22	High-grade glioma, NEC	3	This study (Case 2)
*BRAF*	31,-X,+2,+4q,+7,+9q, +11,+15,+18,+20,+21	High-grade astrocytoma with piloid features	18	In press

## Results

### Case 1

**Figure 1:**
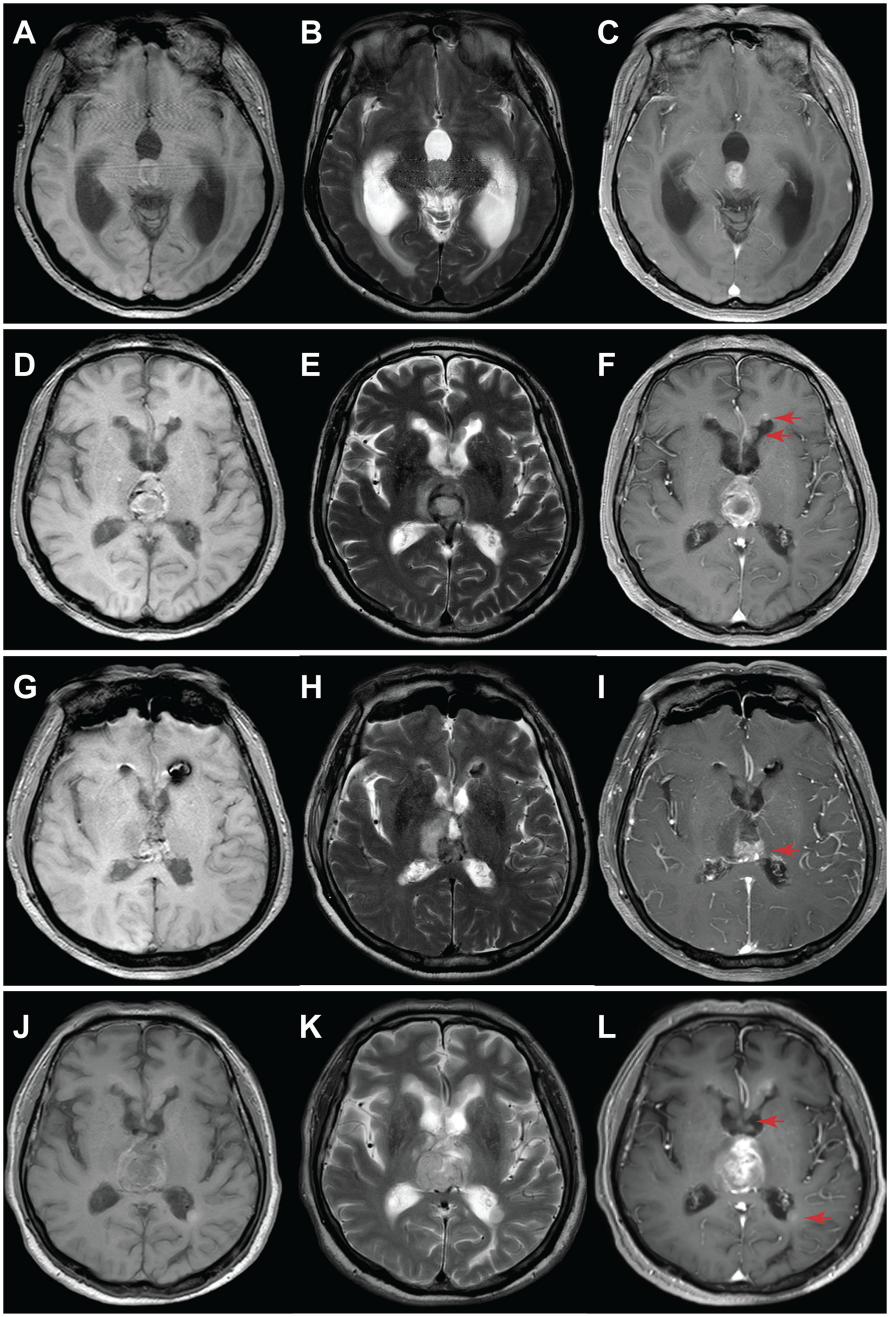
MRI evaluation of tumor progression for Case 1. **(A-C)** Initial diagnosis: A nodular lesion within the third ventricle exhibited slightly T1 hypointense **(A)**, slightly T2 hyperintense **(B)**, and noticeable heterogeneous enhancement **(C)**. Additionally, a significant expansion of the supratentorial ventricular system was observed due to third ventricle exit obstruction. **(D-F)** 3.5 years post-diagnosis: Tumor size increased, accompanied by the appearance of disseminated bilateral lesions within the lateral ventricles (arrow). **(G-I)** 24 hours after surgery: Substantial tumor removal was evident in MRI, with a minor residual segment (arrow) situated near the posterior region of the third ventricle, adjacent to the cerebral vein. **(J-L)** 19 months post-surgery: Tumor recurrence occurred, with an enlarged tumor and increased size and number of lesions within the bilateral lateral ventricles. Left panel: T1-weighted; middle panel: T2-weighted; right panel: T1-weighted contrast-enhanced.

A 59-year-old male presented with unsteady gait and delayed responses for a month, worsening over two weeks before presentation. MRI showed a 13 mm × 15 mm nodular lesion within the third ventricle, obstructing its exit, leading to significant expansion of the supratentorial ventricular system. Contrast-enhanced T1-weighted images exhibited moderate enhancement of the tumor with a relatively regular shape and well-defined margins. T2-weighted images showed no peritumoral edema, and the lesion appeared localized (**Figures 1A–C**). A left lateral ventricle to abdominal cavity cerebrospinal fluid shunt procedure was performed to alleviate cerebral hydrocephalus symptoms. Due to the deep location of the tumor, the patient received radiotherapy by γ knife approach. The tumor relapsed three and a half years later, and bilateral disseminated lesions in the lateral ventricles were observed (**Figures 1D–F**). A surgical procedure was performed using a transcallosal interforniceal approach, which removed most of the tumor within the third ventricle, and postoperative MR imaging demonstrated incomplete tumor resection, with a small residual lesion located posterior of the primary site adjacent to the vein of Galen.(**Figures 1G–I**).

**Figure 2:**
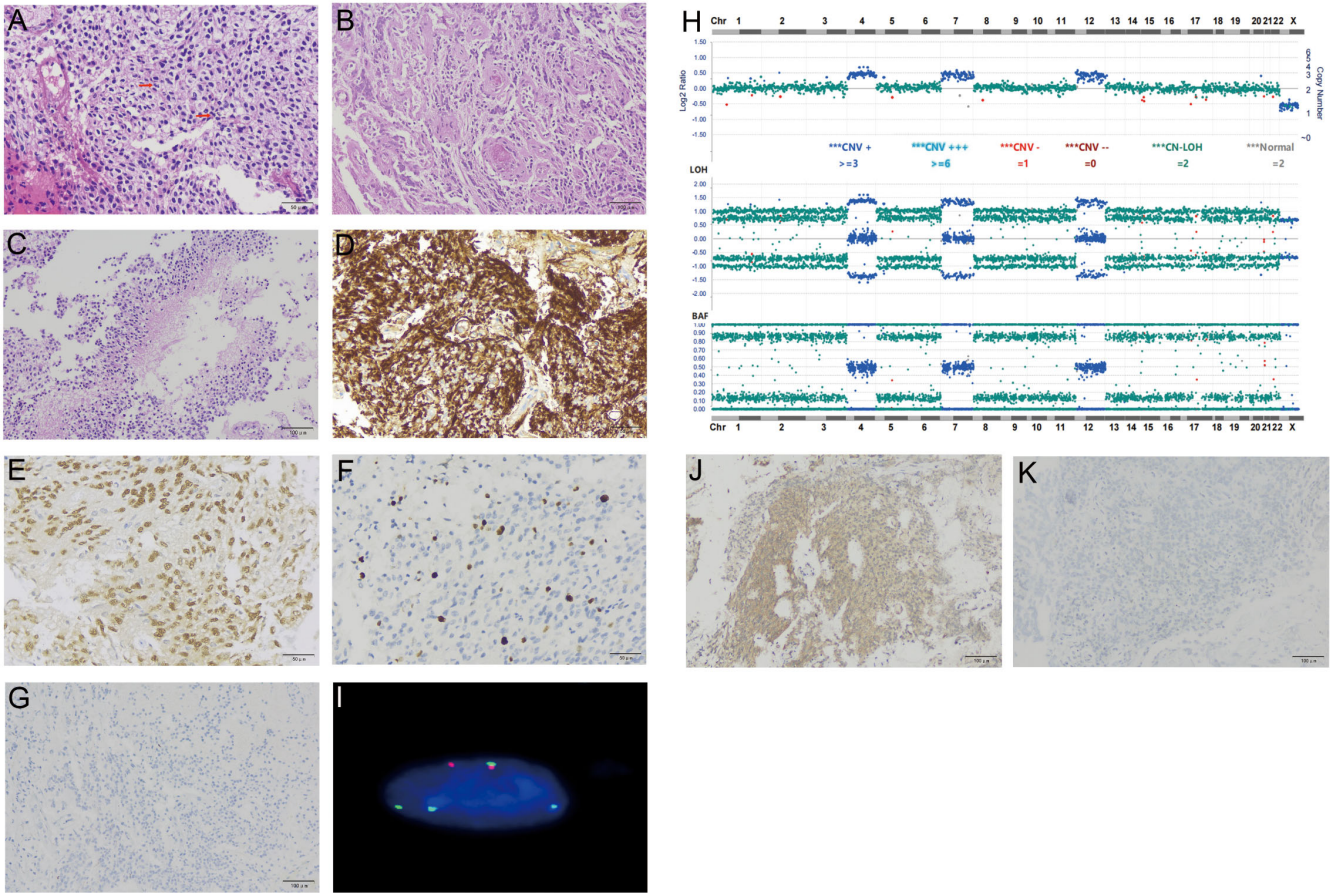
Histological and molecular characterization of the tumor for Case 1. H&E stain of FFPE tumor sections showed increased cellularity and occasional mitoses [arrows, **(A)**], endothelial hyperplasia **(B)**, and palisading necrosis **(C)**. IHC of tumor cells was positive for GFAP **(D)**, Olig2 **(E)**, Ki-67 at 8% **(F)**, and negative for NFP **(G)**. Targeted DNA NGS revealed a near-haploid genome with genome-wide loss of heterozygosity, except for chromosomes 4, 7, and 12 **(H)**. A FISH assay with probes specific for centromeres of chromosomes 7 (green signals) and 8 (red signals) showed tetrasomy 7 and disomy 8, consistent with a duplicated near-haploid genome **(I)**. IHC of tumor cells was positive for phospho p44/42 MAPK [clone # 9102, **(J)**] and negative for MDM2 [clone# IF2, **(K)**].

Hematoxylin and eosin (H&E) staining of formalin-fixed paraffin-embedded (FFPE) sections from tumor tissue showed moderately hypercellularity with nuclear atypia, occasional multinucleated cells, and scattered mitosis. Additionally, regions featuring palisading necrosis and endothelial hyperplasia were identified (**Figures 2A–C**). Mitotic figure scoring found 5 mitoses in 10 high-powered fields. Immunohistochemistry (IHC) was performed on 5-μm tissue sections using BenchMark GX IHC/ISH system from Roche, which was positive for GFAP, Olig-2, MGMT, ATRX, EGFR, and S100, and negative for IDH-1, p53, NEP, NeuN, H3K27M, Syn, CK18, TTF-1, and CD34. Ki-67 staining showed a proliferation index of 8% (**Figures 2D–G**). Based on these observations, a tentative diagnosis of high-grade glioma, NEC was made.

Targeted DNA NGS of 638 cancer genes (**Supplementary Table S1**), coupled with whole-genome single-nucleotide polymorphism (SNP) analysis, was separately performed on tumor DNA from the frozen surgical specimen and germline DNA from peripheral blood. Briefly, approximately 300 ng DNA was fragmented to 200–300 bp, and library preparation was carried out using the Rapid Plus DNA Lib Prep Kit from Illumina (RK20208, ABclonal, USA) following the manufacturer’s instructions. The libraries were incubated with a pool of biotin-labeled bait oligo probes for 16 h, targeted regions were captured with streptavidin beads, amplified by PCR, and sequenced as paired-end 150-bp reads on an Illumina NextSeq 6000 instrument. Reads were aligned to the reference genome (hg19) using BWA-MEM.

Targeted DNA NGS showed a duplicated near-haploid genome with widespread loss of heterozygosity throughout the genome, except for chromosomes 4, 7, and 12, which maintained heterozygosity (**Figure 2H**). Additionally, a single mutation of *FGFR1 c.1966A>G* p.K656E was identified, and IDH was wild type. FISH assays with probes specific to chromosomes 7 and 8 were performed, which showed 4 copies of chromosome 7 and 2 copies of chromosome 8, consistent with the results from the DNA NGS, i.e., a duplicated near-haploid genome (26, X, + 4, +7, +12, duplicated genome) (**Figure 2I**).

Because FGFR1 activation leads to MAPK signaling, IHC with phospho p44/42 MAPK was performed on this tumor, which showed diffuse signals in tumor cells, consistent with MAPK activation (**Figure 2J**). FGFR1 also interacts with MDM2, facilitating the degradation of p53. However, IHC showed no MDM2 expression in our case, suggesting that this pathway is probably nonfunctional (**Figure 2K**).

Adjuvant therapy included a regimen involving both Bevacizumab and Temozolomide. However, after 19 months following surgical resection, a subsequent MRI revealed substantial tumor progression, Compared with the previous MR images, the intraventricular tumor in the third ventricle has markedly increased in size, with invasion of both thalami and new metastatic lesions emerging within the lateral ventricles (**Figures 1J–L**). The patient passed away shortly after due to a stroke, which was precipitated by alcohol consumption.

### Case 2

**Figure 3:**
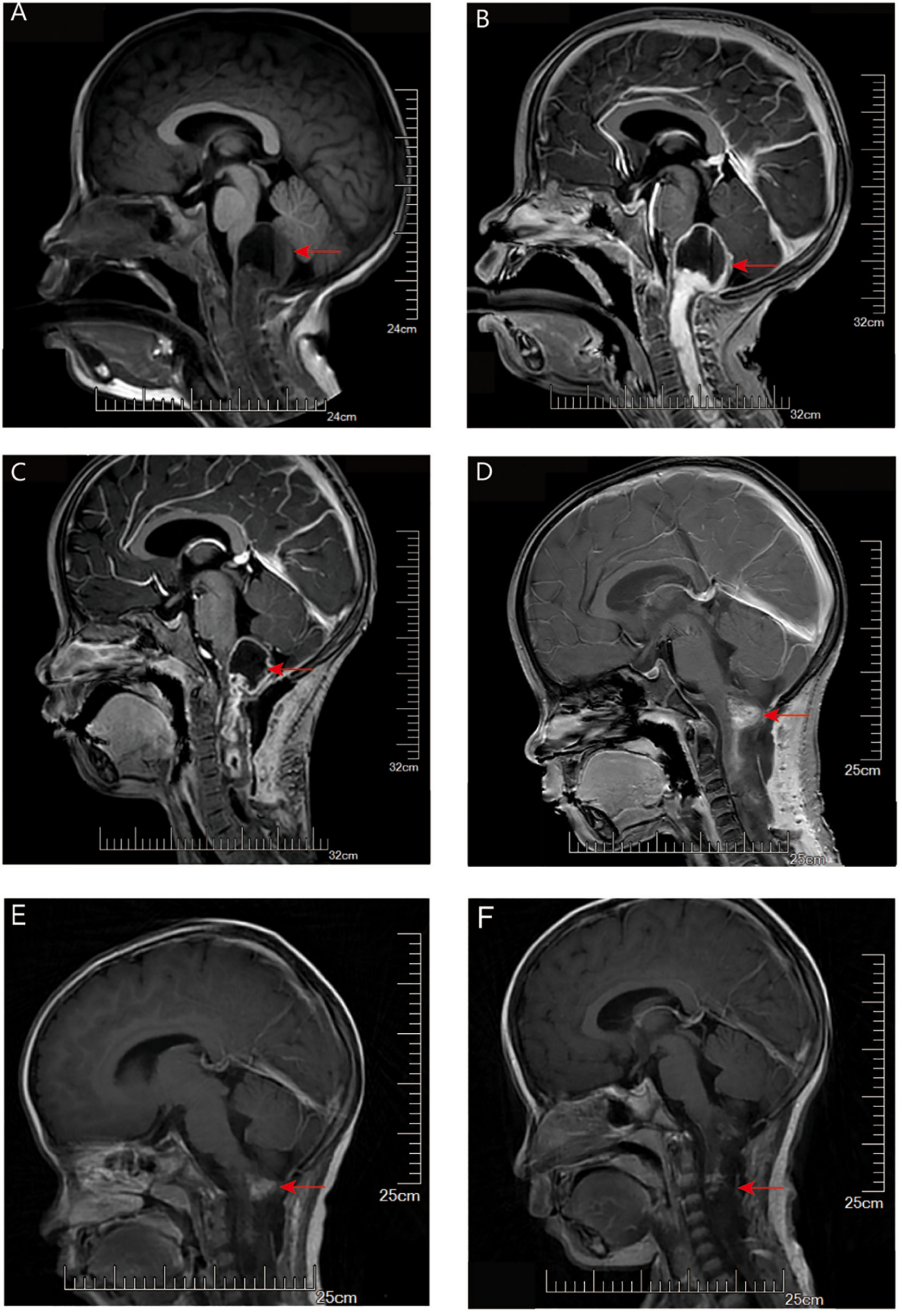
MRI evaluation of tumor for Case 2. **(A)** T1-weighted imaging (T1WI): A mixed cystic-solid mass is seen in the pineal region. The cystic component extends posteriorly beyond the pineal body and demonstrates a fluid-fluid level. There is marked compression of the surrounding peri-mesencephalic cisterns and subarachnoid space below the tentorium. **(B)** Contrast-enhanced T1WI: The solid component of the tumor shows significant contrast enhancement, while the cystic portion remains non-enhancing. **(C)** Contrast-enhanced T1WI one month after surgery (before chemotherapy): The tumor has decreased in size. Areas of necrosis have increased compared to baseline, and the subarachnoid space below the tentorium appears slightly expanded. **(D–F)** Contrast-enhanced T1WI images obtained at 12 weeks **(D)**, 3 months **(E)**, and 5 months **(F)** after the initiation of chemotherapy showed progressive tumor shrinkage with reduction in contrast enhancement.

A 3-year-old boy presented with intermittent crying and discomfort for half a month, occasional dry heaving, and a recent onset of numbness and weakness in both hands. MRI showed a cystic mass in the posterior fossa with a fluid-fluid level, measuring approximately 28 mm x 23 mm, compressing the medulla oblongata, adjacent cerebellum, and fourth ventricle (**Figures 3A, B**). A brainstem tumor resection was performed. Postoperative MR imaging demonstrated an incomplete tumor resection, with predominantly cystic, round-shaped lesion with mixed signal intensity is visible in the posterior fossa, slightly larger than previously observed. On contrast-enhanced imaging, the lesion exhibits a distinct ring-like enhancement at its margins. Extensive areas of abnormal signal intensity are noted in the medulla oblongata, cervical spinal cord, and upper thoracic spinal cord. One month after surgery and before the initiation of chemotherapy, follow-up MRI showed that the areas of necrosis had increased compared to baseline, and the subarachnoid space below the tentorium slightly expanded (**Figure 3C**).

**Figure 4:**
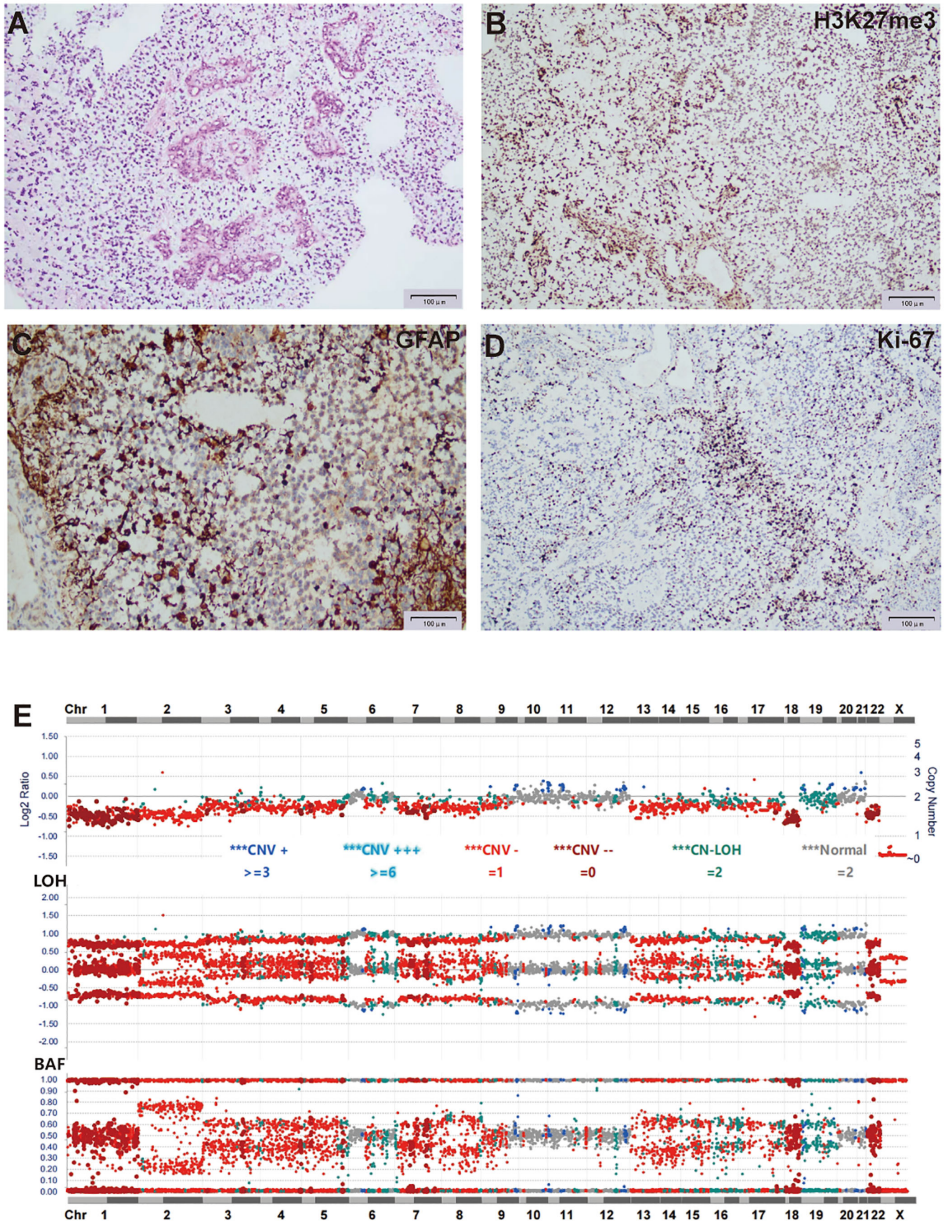
Histological and molecular characterization of the tumor for case 2. **(A)** H&E stain of FFPE tumor sections showed moderate cellularity, with hyperchromatic nuclei, nuclear atypia, and prominent microvascular proliferation. **(B-D)** IHC of tumor cells was positive for H3K27me3 **(B)**, GFAP **(C)**, and Ki-67 at 40% **(D)**. **(E)** Targeted DNA NGS showed widespread chromosome loss.

H&E staining of FFPE sections from tumor tissue showed moderate cellularity, with hyperchromatic nuclei, nuclear atypia, and prominent microvascular proliferation (**Figures 4A, B**). IHC was positive for Olig-2, BRG1, INI1, GFAP, H3K27me3, and S100, and negative for H3K27M, EMA, and NeuN. Ki-67 staining showed a proliferation index of 40% (**Figures 4C, D**). Based on these observations, a tentative diagnosis of high-grade glioma, NEC was made.

Targeted DNA NGS of the tumor tissue identified two *FGFR1* mutations, *c.1972A>C* p.T658P and *c.1968G>C* p.K656N, both located within the FGFR1 kinase domain and representing the sole pathogenic mutations. Additionally, copy number analysis showed extensive chromosomal loss, with heterozygosity retained in only six chromosomes (28, -X, +6, +10, +11, +12, +21, +22) (**Figure 4E**).

Postoperative adjuvant chemotherapy consisted of an induction phase followed by a maintenance phase designed to optimize chemotherapy efficacy while allowing for hematologic recovery. For induction, vincristine (VCR) at a dose of 1.5 mg/m² and cyclophosphamide (CP) at 175 mg/m² intravenously, were administered on days 0, 7, 14, 21, 28, 35, 42, 49, 56, and 63, spanning approximately 12 weeks. Maintenance therapy began once the complete blood count (CBC) recovered to an absolute neutrophil count (ANC) greater than 1,000 and platelets exceeding 100,000, involving the same VCR and CP dosages on days 0, 7, 14, and 21 of each 42/0-day cycle, continued for up to 8 cycles. Serial MRI scans demonstrated a continued decrease in tumor size, with near-complete resolution of contrast enhancement (**Figures 3D–F**).

To further characterize both cases, whole-genome methylation profiling of tumor DNA was performed using the Infinium MethylationEPIC v2 BeadChip (Illumina, USA). Bisulfite conversion was performed using the Zymo EZ Methylation Kit (Zymo Research, Irvine, USA), following the manufacturer’s protocol. DNA was then treated with the Infinium HD FFPE Restore Kit (Illumina, USA), hybridized to the BeadChips, and scanned using the iScan system (Illumina, USA). Raw IDAT files were uploaded and analyzed using the Brain Tumor Classifier v12.8. However, based on the stringent calibrated classifier score cutoff of ≥0.9, neither case could be confidently assigned to a specific methylation class. Case 1 yielded the highest match score of 0.45 to the “Low-Grade Glial/Glioneuronal/Neuroepithelial Tumors” class, while Case 2 reached a score of 0.87 for the same class. Furthermore, an unsupervised t-SNE analysis revealed that Case 1 was positioned at the border between low-grade glioma and schwannoma, while Case 2 was located in the peripheral region of low-grade glioma, with neither showing high overlap with the reference cohort. In a prospective cohort study, 12% of tumors (127/1,104) could not be assigned to a DNA methylation category using a strict calibration classifier score threshold of ≥0.9 (6). In addition, the histologic features of these cases are not consistent with a low-grade glioma. Together, these findings suggest that such cases may represent previously unrecognized rare novel molecular entities.

## Discussion

The classification of this tumor presents challenges based on both histology and genomics, and the whole-genome methylation study was unsuccessful in assigning it to a specific methylation class.

The presence of endothelial hyperplasia and necrosis in Case 1 aligns with a CNS WHO grade 4 tumor. However, the wild-type *IDH1/2* contradicts compatibility with a high-grade astrocytoma or oligodendroglioma, and the absence of *TERT* mutation and *EGFR* amplification does not align well with glioblastoma. While the haploid genome is characteristic of giant cell glioblastoma (7), the morphology in both cases does not fulfill the WHO classification criteria, which require multinucleated giant cells to be a predominant histopathological feature. Additionally, giant cell glioblastoma is frequently associated with *TP53* mutations, which are absent in this cases. In addition, the lack of NFP expression suggests the absence of entrapped neurons within the tumor, which argues against a diffuse glioma (**Figure 2G**). Collectively, a tentative diagnosis of high-grade glioma, NEC was assigned to the tumors. Unfortunately, due to the primary tumor’s deep location, we were unable to obtain a specimen of the primary tumor from Case 1. However, given the presence of a solitary *FGFR1* mutation and the circumscribed nature of the relapsed tumor, our suspicion points toward a pilocytic astrocytoma for the primary tumor.

Case 2 showed moderate cellularity, with hyperchromatic nuclei, nuclear atypia, and prominent microvascular proliferation, features consistent with a high-grade glioma. Genomically, the tumor is *H3 K27/G34* wild-type and shows no alterations in MYCN, MYC, or tyrosine kinase receptors, which are commonly seen in pediatric high-grade gliomas. Thus, based on histology, genomics, and methylation profiling, this tumor does not fit a clearly defined WHO CNS tumor classification. Similar to Case 1, the tumor was designated as high-grade glioma, NEC.

Polyploidy is a common phenomenon in tumors and can be observed in most tumor types. In contrast, haploidy is relatively rare and has been reported in only a few tumors. These include giant cell glioblastoma, inflammatory leiomyosarcoma, oncocytic follicular thyroid carcinoma/Hürthle cell carcinoma, B-cell acute lymphoblastic leukemia (B-ALL), and peripheral chondrosarcoma (8–10). Near-haploid tumors often show a consistent loss of specific chromosomes that harbor known tumor suppressor genes. These include chromosome 9 (*CDKN2A/B*), chromosome 10 (*PTEN*), chromosome 13 (*RB1*), chromosome 17 (*TP53* and *NF1*), and chromosome 22 (*NF2*). Additionally, certain chromosomes are retained (disomies) in a tumor type-specific manner. For instance, giant cell glioblastoma retains disomic 7, inflammatory leiomyosarcoma retains disomic 5 and 22, oncocytic follicular thyroid carcinoma/Hürthle cell carcinoma retains disomic 5, 7, and 12, B-ALL retains disomic 8, 10, 14, 18, 21, X, and Y, and peripheral chondrosarcoma retains disomic 7. The retention of specific chromosomes suggests the presence of critical survival genes located in these regions, which may vary depending on the cell type. In our Case 1, the retention of two copies of chromosomes 4, 7, and 12 (disomies in the haploid genome, tetrasomy in the duplicated genome). In the previously reported *NF1*-mutant A-PXA, two copies of chromosomes 4, 5, 7, 12, and 16 were retained, sharing disomies 4, 7, and 12 with our case. Similarly, in the 5th case involving the transformation of pilocytic astrocytoma, retention of two copies of chromosomes 4q and 7 was also observed (**Table 1**). Notably, important oncogenes involved in brain tumor development are located on these chromosomes, such as *PDGFR1* and *FGFR2* on chromosome 4, *EGFR* and *MET* on chromosome 7, and *CDK4* and *MDM2* on chromosome 12. Further characterization of these critical oncogenes could provide valuable insights into tumorigenesis and potentially uncover novel therapeutic approaches by targeting those survival-essential genes.

**Figure 5:**
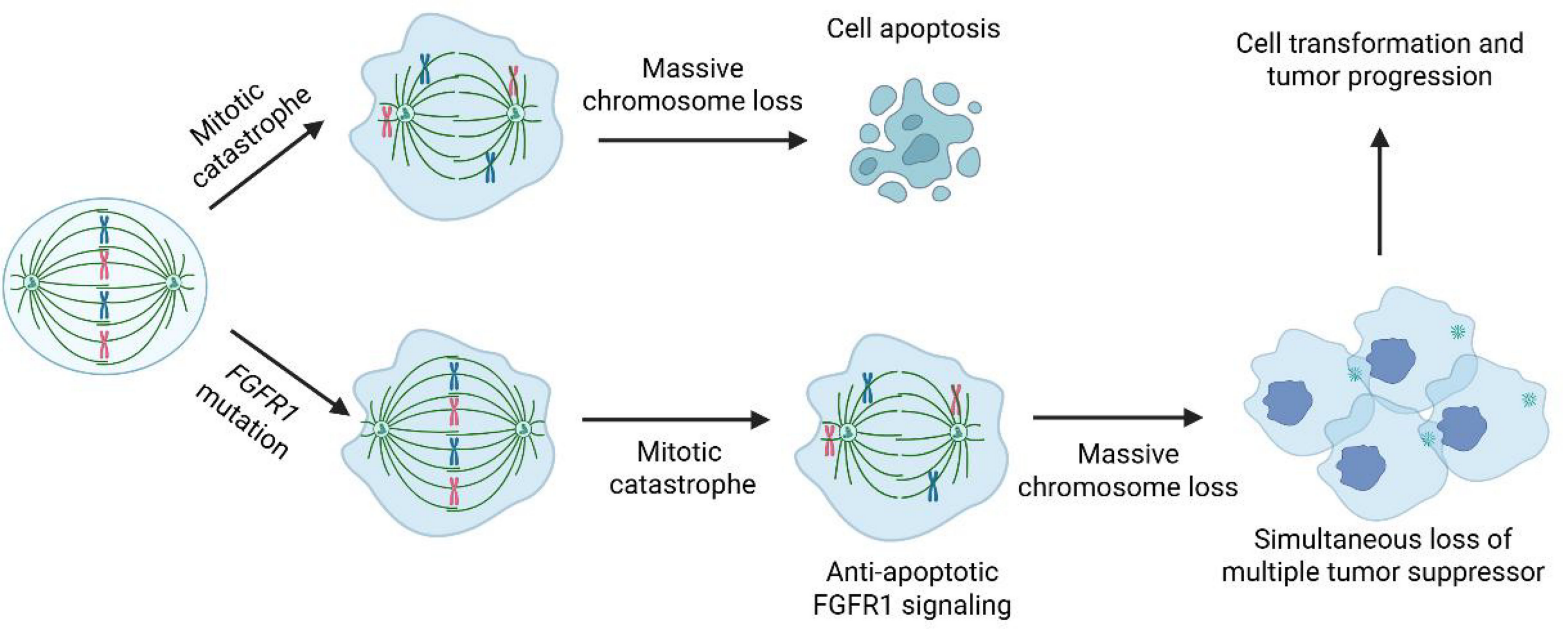
Hypothetical model illustrating mitotic catastrophe-induced cell transformation and tumor progression.

Complex genomic rearrangements typically occur through mechanisms such as chromosomal break-fusion bridges, monosomy, or chromosomal fragmentation. These mechanisms lead to genomic instability, resulting in a large number of structural variations, including oncogene activation, tumor suppressor gene inactivation, or alterations in gene regulatory networks. Complex genomic rearrangements may lead to the amplification of oncogenes (e.g., *EGFR, PDGFRA*) or the loss of tumor suppressor genes (e.g., *TP53, RB1*). For example, *EGFRvIII* (a truncated variant of *EGFR*) is a common amplification product in glioblastoma and is associated with increased tumor invasiveness. Complex genomic rearrangements can generate oncogenic fusion genes, such as the *FGFR3-TACC3* fusion, which has been reported to drive tumor cell proliferation and invasion in gliomas. Complex genomic rearrangements increase genomic instability, accelerate mutation accumulation, and promote tumor heterogeneity and adaptive evolution. Complex genomic rearrangements may also disrupt gene regulatory elements (e.g., enhancers or suppressors), leading to abnormal gene expression and affecting tumor cell proliferation, differentiation, or apoptosis. Interestingly, this subgroup of high-grade gliomas is characterized by extensive chromosomal loss rather than complex structural rearrangements. Such massive chromosome loss can occur during mitotic catastrophe (11), resulting in widespread numerical chromosomal abnormalities arising from a single event. Typically, near-haploid cells do not survive due to the severe depletion of genomic material. However, in the presence of FGFR1 activation, which is known to promote anti-apoptotic signaling, cell survival may be facilitated. Moreover, the concurrent loss of multiple chromosomes can lead to the simultaneous inactivation of several tumor suppressor genes, potentially driving tumor progression and malignant transformation (**Figure 5**).

FGFRs play a significant role in cancer, including brain tumors. FGFR inhibitors have been developed, including three FDA-approved drugs. Erdafitinib is approved for locally advanced or metastatic urothelial carcinoma with susceptible *FGFR3* or *FGFR2* genetic alterations. Infigratinib and pemigatinib are approved for cholangiocarcinoma with an *FGFR2* rearrangement. FGFR inhibitors have demonstrated promising anti-tumor activity in preclinical *in vitro* and *in vivo* models of brain tumors. Additionally, they have shown encouraging results in clinical trials (10). Ongoing research focuses on the efficacy of various FGFR inhibitors, both as single agents and in combination with other treatment modalities, in brain tumors. These targeted therapies offer the potential for personalized treatment strategies in patients with *FGFR*-altered brain tumors, potentially leading to improved therapeutic options and outcomes.

In summary, we present two cases of high-grade glioma, NEC characterized by a distinct genomic profile: an *FGFR1* activation mutation and massive chromosome loss. Our hypothesis suggests that the *FGFR1* mutation acts as an initiating event, while the widespread chromosome loss serves as a secondary alteration that plays a crucial role in driving malignant transformation (**Figure 5**). Similar genomic alterations, specifically a mutation in one of the *MAPK* genes combined with a near-haploid genome, have been observed in 3 additional high-grade gliomas (**Table 1**). The potential role of haploid formation, likely stemming from a catastrophic mitotic error, as an oncogenic mechanism driving malignant transformation remains highly speculative at this point. However, documenting these cases is significant for future evaluation and research.

## Data Availability

The raw data supporting the conclusions of this article will be made available by the authors, without undue reservation.
