# Correction: Case Report: *FGFR1* mutation and massive chromosome loss drive malignant transformation of low-grade gliomas

**DOI:** 10.3389/fonc.2025.1748919

**Published:** 2025-12-03

**Authors:** Ruze Tang, Yanming Chen, Dong Wan, Yongjun Xiang, Si Chen, Huafei Chen, Xiaoxiao Dai, Rong Rong, Sheng Xiao, Qing Lan, Hangzhou Wang, Shungen Huang

**Affiliations:** 1 Department of General Surgery, Children’s Hospital of Soochow University, Suzhou, China; 2 Department of Neurosurgery, The Second Affiliated Hospital of Soochow University, Suzhou, China; 3 Advanced Molecular Pathology Institute of Soochow University and SANO, Suzhou, China; 4 Suzhou SANO Precision Medicine Ltd, SANO Medical Laboratories, Suzhou, China; 5 Department of Neurosurgery, Children’s Hospital of Soochow University, Suzhou, China; 6 Department of Pathology, The Second Affiliated Hospital of Soochow University, Suzhou, China; 7 Department of Biological Sciences, Xi An Jiaotong-Liverpool University, Suzhou, China

**Keywords:** *FGFR1*, haploidy, glioma, malignant transformation, methylation

Affiliation “Department of Neurosurgery, The Second Affiliated Hospital of Soochow University, Suzhou, China” was omitted for author “Qing Lan”. This affiliation has now been added for author “Qing Lan”.

Author “Qing Lan” was erroneously assigned to affiliation “Department of General Surgery, Children’s Hospital of Soochow University, Suzhou, China”. This affiliation has now been removed for author “Qing Lan”.

The **Conflict of interest statement** has been correct to “Authors DW, SC, HC, and SX were employed by Suzhou SANO Precision Medicine Ltd.

The remaining authors declare that the research was conducted in the absence of any commercial or financial relationships that could be construed as a potential conflict of interest”.

The original version of this article has been updated.

